# Influence of temperature, salinity and Mg^2+^:Ca^2+^ ratio on microbially-mediated formation of Mg-rich carbonates by *Virgibacillus* strains isolated from a sabkha environment

**DOI:** 10.1038/s41598-019-56144-0

**Published:** 2019-12-23

**Authors:** Zulfa Ali Al Disi, Tomaso R. R. Bontognali, Samir Jaoua, Essam Attia, Hamad Al Saad Al-Kuwari, Nabil Zouari

**Affiliations:** 1 Department of Biological & Environmental Sciences, College of Arts Sciences, Qatar University, P.O. Box 2713, Doha, Qatar; 2 ETH Zürich, Department of Earth Sciences, Sonneggstrasse 5, 8092 ETH Zürich, Zürich, Switzerland; 3 Space Exploration Institute (SPACE-X), 68 Faubourg de l’Hopital, 2000 Neuchatel, Switzerland; 4 University of Basel, Department of Environmental Sciences, Klingelbergstrasse 27, Basel, Switzerland; 5 Central Laboratory Unit, Qatar University, P.O. Box 2713, Doha, Qatar; 6 Environmental Science Centre, Qatar University, P.O. Box 2713, Doha, Qatar

**Keywords:** Chemical ecology, Carbon cycle

## Abstract

Studies have demonstrated that microbes facilitate the incorporation of Mg^2+^ into carbonate minerals, leading to the formation of potential dolomite precursors. Most microbes that are capable of mediating Mg-rich carbonates have been isolated from evaporitic environments in which temperature and salinity are higher than those of average marine environments. However, how such physicochemical factors affect and concur with microbial activity influencing mineral precipitation remains poorly constrained. Here, we report the results of laboratory precipitation experiments using two mineral-forming *Virgibacillus* strains and one non-mineral-forming strain of *Bacillus licheniformis*, all isolated from the Dohat Faishakh sabkha in Qatar. They were grown under different combinations of temperature (20°, 30°, 40 °C), salinity (3.5, 7.5, 10 NaCl %w/v), and Mg^2+^:Ca^2+^ ratios (1:1, 6:1 and 12:1). Our results show that the incorporation of Mg^2+^ into the carbonate minerals is significantly affected by all of the three tested factors. With a Mg^2+^:Ca^2+^ ratio of 1, no Mg-rich carbonates formed during the experiments. With a Mg^2+^:Ca^2+^ ratios of 6 and 12, multivariate analysis indicates that temperature has the highest impact followed by salinity and Mg^2+^:Ca^2+^ ratio. The outcome of this study suggests that warm and saline environments are particularly favourable for microbially mediated formation of Mg-rich carbonates and provides new insight for interpreting ancient dolomite formations.

## Introduction

Studies conducted during the last 20 years in modern natural environments and in the laboratory have shown that some microorganisms promote the formation of Mg-rich carbonate minerals, including phases that may be precursor of ordered dolomite (e.g.^1–8^). These studies are relevant to understand the origin of sedimentary dolomite^9–11^, a mineral that is abundant in ancient sedimentary rocks, but whose formation may result from substantially different and not fully understood processes^12,13^. Because sedimentary sequences rich in dolomite are often studied for paleoclimatic and paleoenvironmental reconstructions and because dolomite comprises many economically important gas and oil reservoirs^14^ much effort has been, and is still being invested in understanding the processes that leads to its formation.

Microorganisms that have been shown to catalyse the incorporation of Mg into carbonate minerals at low temperatures include sulfate reducers^4,11,15–18^, methanogens^19,20^, and aerobic heterotrophs^5,21^. The exact mechanims by which these microorganisms mediate the formation of Mg-rich carbonate minerals has not been fully resolved. The metabolism of mineral-mediating microorganisms often leads to an increase in alkalinity and pH that favor supersaturation and consequent precipitation of carbonate minerals^12,16,18,22^.

However, conditions of supersaturation with respect to dolomite are insufficent to explain its precipitation at low temeperature, which is likely inhibited by kinetic factors. An increasing number of studies suggest that microbially-produced organic molecules may play a key role in overcoming such kinetic barriers^23–25^. A recurring and plausible hypothesis posits that some functional groups (e.g., carboxyl groups) present in extracellular polymeric substances (EPS) or in the cell wall adsorb ions (e.g., Mg^2+^), creating a supersaturated local environment^26,27^ and also reducing the activation energy necessary for the initiation of crystal growth (e.g., by dehydrating Mg^2+^)^24,25,28^.

Most of the microbial “protagonists” in the studies mentioned above were isolated from environments characterized by high temperature (average annual 20 °C or above), salinity (twice seawater or more) and a high Mg^2+^: Ca^2+^ ratio (above 8)^2,5,21,29^. From a purely physicochemical rather than geobiological perspective, all these factors have been proposed as important –although not necessarily essential^30^ – for the formation of carbonate minerals^31–33^. Temperature is known to promote the incorporation of Mg into carbonates^34,35^. Salinity and the increased concentration of Mg^2+^ (and the Mg^2+^:Ca^2+^ ratio) can also cause supersaturation with respect to dolomite^36^. It is therefore likely that a combination of abiotic parameters determines the optimum conditions for the formation of microbially mediated Mg-rich carbonates^37^. For instance, sulfate reducing bacteria are virtually ubiquitous in the marine realm^38^, but their ability to mediate the formation of dolomite may be expressed only in a hypersaline environment in waters that have a high Mg^2+^:Ca^2+^ ratio (i.e., exceeding the ratio of 5 that characterizes modern marine conditions)^2,4,11,39^. Indeed, extensive evaporation leads to the precipitation of aragonite and subsequently of gypsum, which results in Ca^2+^ removal from solution and an increased Mg^2+^: Ca^2+^ ratio^29^. On the other hand, in evaporitic environments the positive effect of a high Mg^2+^:Ca^2+^ ratio might be thwarted by a salinity that is so high to inhibit microbial growth^6,40^. Knowing the optimal conditions for microbially mediated formation of very high Mg calcite —often considered as a potential dolomite precursor^41^— would be very helpful for interpreting the paleoenvironment associated with this type of dolomite in ancient sedimentary sequences. Because microbial species react differently to changes in physiochemical parameters^42^, the identification of optimal conditions for the microbial formation of Mg-rich carbonates is very challenging and caution is required before generalized conclusions can be made. There is as yet, a very limited number of studies evaluating the combined effect of several environmental factors on microbial (eco)physiology^33,43,44^.

To contribute in understanding such a complex process, here, the interactive effects of temperature, salinity and Mg^2+^:Ca^2+^ ratio on microbially mediated formation of Mg-rich carbonates at aerobic conditions by the bacterium *Virgibacillus* were investigated. Two strains of this bacterium, known to mediate the formation of Mg-rich carbonates^5^ and one non-mineral forming bacterial *Bacillus*, all previously isolated from the Dohat Faishakh sabkha (a modern dolomite-forming environment located in Qatar) were used for the experiments.

## Results

### Impact of NaCl (%w/v), temperature, and Mg^2+^:Ca^2+^ ratios on the growth of *Virgibacillus* and crystal formation

In order to investigate the impact of the three abiotic factors on the growth of *Virgibacillus* and crystal formation, two strains previously isolated, identified, and characterized as high magnesium calcite (HMC) forming strains^5^, were compared to a non-crystal producing bacterium *Bacillus lechinforms* (Table 1). The earliest visible growth began after 24 h at a 40 °C incubation temperature, while the precipitation often started 1–3 days after extensive growth was observed. Extensive growth followed by precipitation started earlier in cultures with the lowest-tested Mg^2+^:Ca^2+^ ratio of 1. However by the end of incubation period, the total number of crystals formed was lower in cultures with a Mg^2+^:Ca^2+^ ratio of 1 than in cultures with Mg^2+^:Ca^2^ ratios of 6 and 12. All studied strains caused an increase in pH of the artificial growth medium (i.e., from 7.0 to 8.5), regardless of their capability to mediate mineral formation. No indications about the mineral composition could be drawn at this stage. No mineral formation was observed in any negative control cultures (i.e., non-crystal producing bacteria, autoclaved cells, uninoculated media, and modified MD medium with no acetate salts).

**Table 1:** Results of culture experiments for mineral forming strains at different temperatures and NaCl (%w/v) observed by optical microscope (40×).

Temperature (°C)	NaCl (%w/v)	Mg^2+^: Ca^2+^	Initial growth (d)	Initial precipitation (d)**	Extensive precipitation (d)**	Amount of crystals formed*
20	3.5	1	3	5	8	++
20	7.5	1	3	5	10	++
20	10	1	3	5	10	+
20	3.5	6, 12	3	5	10	+++
20	7.5	6, 12	3	5	10–12	+++
20	10	6, 12	3	7	10–12	+++
30	3.5	1	2	3	5	+++
30	7.5	1	2	3	5	++
30	10	1	2–3	5	7	++
30	3.5	6, 12	2	3	5	+++
30	7.5	6, 12	2	3	5	+++
30	10	6, 12	2–3	5	7	+++
40	3.5	1	1	2	3	++
40	7.5	1	1	2	5	++
40	10	1	1–2	3	5	++
40	3.5	6, 12	1	2	3	++++
40	7.5	6, 12	1	2	5	++++
40	10	6, 12	1–2	3	5	++++

Adapted from corresponding PhD Dissertation^71^.

^*^Qualitative estimation, average number of crystals/mm^2^, ^+^: 1–14, ++: 15–49, +++: 50–99, ++++: >100.

^**^Only for the mineral forming strains DF112 and DF2141.

Number of view fields n = 10, Standard deviation < 5%.

### Impact of NaCl (%w/v), temperature, and Mg^2+^:Ca^2+^ ratios on morphology and composition of crystals

**Figure 1:**
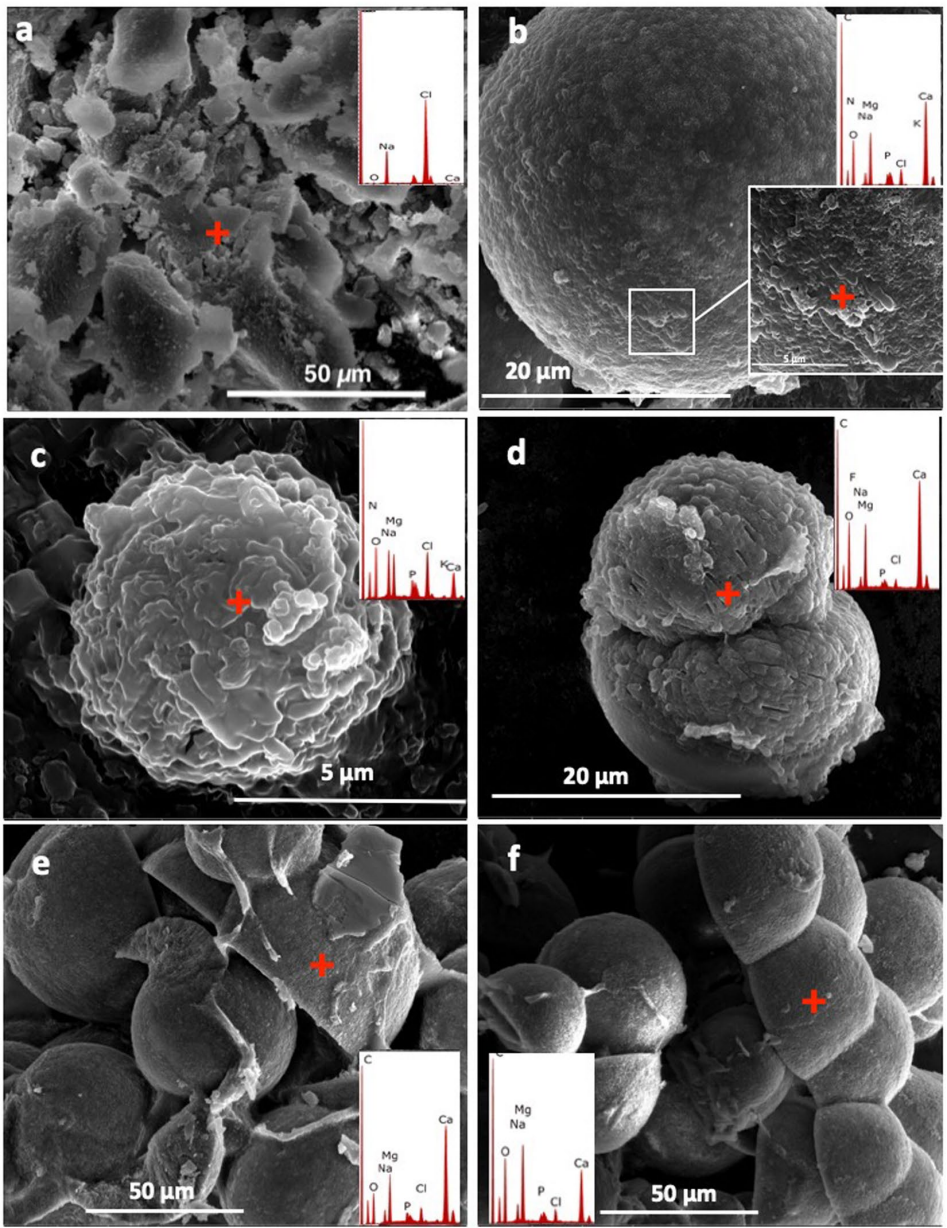
SEM/EDS investigations of minerals recovered from DF141, DF112 and DF2141 cultures. (**a**) Negative control DF141, elemental analysis indicates the presence of sodium chloride, (**b**) calcium carbonate crystals formed in DF112 cultures at 20 °C; close-up showing bacterial cells embedded in the calcium carbonate crystal. (**c**) Mineralized bacterial cells. (**d**) High-magnesium carbonate crystal recovered from DF112 culture at 30 °C, showing moulds of bacterial cells. Mixture of Mg-rich carbonate crystals formed in DF2141 cultures at (**e**) 30 °C and (**f**) 40 °C, showing similar morphologies but with smaller crystals and more incorporation of Mg^2+^ at 40 °C. C: calcite, H: halite. Modified from corresponding PhD Dissertation^71^.

Scanning electron microscopy/energy-dispersive X-ray spectroscopy (SEM/EDS) investigations of the minerals formed in the DF112 and DF2141 cultures incubated at various NaCl (%w/v), temperatures, and Mg^2+^:Ca^2+^ ratios showed the presence of peloids composed of carbonate minerals (Fig. 1a–d). The dominant morphologies were spheres with a rough, grainy surface with a size ranging from 2 μm to100 μm. Fewer dumbbell- and cap-shaped peloids were also observed. Many surfaces of spheres showed embedded bacterial cells, bacterial moulds, and nanoparticles.

**Figure 2:**
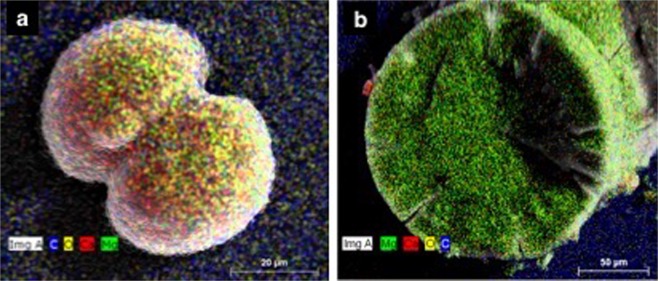
Representative EDS elemental maps showing the distribution of elements in crystals recovered from DF112 and DF241 pure cultures. (**a**) High magnesium calcite crystal. (**b**) Hydromagnesite crystal.

The EDS spectra and EDS elemental mapping of the peloids indicated the presence of C, O, P, Ca^2+^, and Mg^2+^ but at various ratios (Figs. 1, 2). The size and composition of the peloids varied for each pure culture, and between different abiotic conditions. Larger spheres and cap-shaped peloids were observed when the cultures were incubated at lower temperatures (Fig. 1(e,f)). No carbonate minerals were formed in the DF141 cultures (Fig. 1(a)).

**Figure 3:**
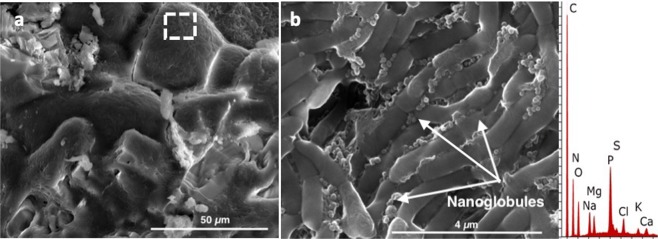
(**a**) Virgibacillus cells covering a carbonate mineral formed during the experiments. (**b**) Close-up image showing nanoglobules aggregates on the outer bacterial cells. EDS results indicate the bulk elemental composition of (**b**). Adapted from corresponding PhD Dissertation^71^.

Moreover, it was possible to detect bacterial cells covering the minerals formed (Fig. 3(a)), and a close-up image revealed an aggregation of nanoglobules on the outer bacterial cells (Fig. 3(b)).

**Figure 4:**
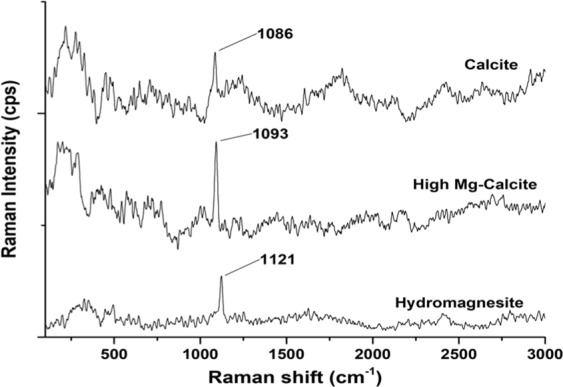
Raman spectra of different types of crystal formed in *Virgibacillus* culture experiments compared to that of calcite, dolomite, and hydromagnesite standards.

The observed bands in Raman shift spectroscopy confirm the occurrence of calcite, high magnesium calcite and hydromagnesite (Fig. 4). Based on comparisons with available reference standards (downloaded from http://rruff.info/) and data reported in the literature, the ν_1_, *symmetric stretching mode* of the carbonate observed at 1086 corresponds to calcite^45^, the increase in the carbonate v_1_ peak position at 1093 may be attributed to an increase in magnesium content^46^. The 1121 cm^−1^ Raman band is consistent with the v1 band of hydromagnesite, which is characterized by frequencies ranging from 1117 to 1120 cm^−1^ ^47^.

**Figure 5:**
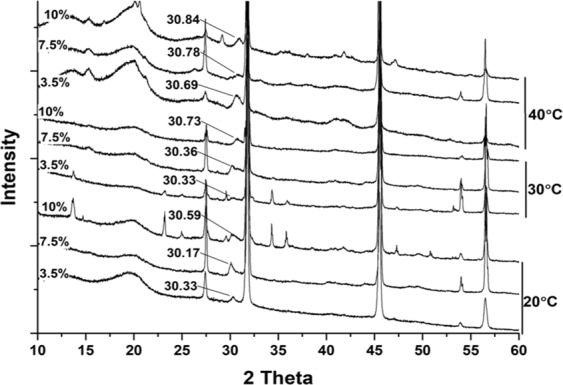
Representative X-ray diffraction patterns of minerals recovered from *Virgibacillus* cultures using media with different salinity levels, and temperatures and with a Mg^2+^: Ca^2+^ ratio of 6. Adapted from corresponding PhD Dissertation^71^.

**Figure 6:**
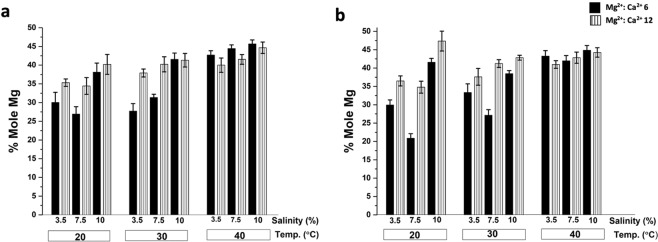
Effect of temperature, salinity and Mg^2+^: Ca^2+^ ratio on the incorporation of Mg (%Mol Mg) into the carbonate minerals recovered from pure cultures of: DF112 (**a**) and DF 2141 (**b**), at Mg^2+^: Ca^2+^ 6 and 12. Adapted from corresponding PhD dissertation^71^.

The XRD analysis of the recovered bulk minerals revealed the presence of very high-magnesium calcite (VHMC) with a variable mol% of Mg (i.e., 26.59 ± 1.65 to 47.37 ± 2.71) in cultures that had initial Mg^2+^:Ca^2+^ ratios of 6 and 12 (Fig. 5, Table 2 and Fig. 6).

**Table 2:** Mg Mol% of the crystals recovered from different cultures.

Strain	Medium	Mg^2+^: Ca^2+^	NaCl (%w/v)	Temperature (°C)	Mg Mol%	Ref.
DF112	MD4	6	3.5	20	30.02 ± 2.60	Ref. ^72^
	MD5	6	7.5	20	26.59 ± 1.65	
	MD6	6	10	20	38.45 ± 1.35	
	MD4	6	3.5	30	27.70 ± 2.00	
	MD5	6	7.5	30	31.63 ± 0.62	
	MD6	6	10	30	41.52 ± 1.71	
	MD4	6	3.5	40	42.17 ± 2.02	
	MD5	6	7.5	40	44.43 ± 0.97	
	MD6	6	10	40	46.82 ± 1.14	
	MD7	12	3.5	20	35.32 ± 0.99	This work
	MD8	12	7.5	20	34.44 ± 2.24	
	MD9	12	10	20	40.21 ± 2.65	
	MD7	12	3.5	30	38.21 ± 1.96	
	MD8	12	7.5	30	40.23 ± 2.03	
	MD9	12	10	30	41.52 ± 1.80	
	MD7	12	3.5	40	40.01 ± 1.91	
	MD8	12	7.5	40	41.41 ± 1.31	
	MD9	12	10	40	44.64 ± 1.52	
DF2141	MD4	6	3.5	20	29.93 ± 1.38	Ref. ^72^
	MD5	6	7.5	20	20.87 ± 1.25	
	MD6	6	10	20	41.60 ± 1.06	
	MD4	6	3.5	30	33.34 ± 2.38	
	MD5	6	7.5	30	27.15 ± 1.51	
	MD6	6	10	30	38.49 ± 0.82	
	MD4	6	3.5	40	43.25 ± 1.52	
	MD5	6	7.5	40	41.95 ± 1.46	
	MD6	6	10	40	44.85 ± 1.29	
	MD7	12	3.5	20	36.49 ± 1.36	This work
	MD8	12	7.5	20	34.12 ± 2.21	
	MD9	12	10	20	47.37 ± 2.71	
	MD7	12	3.5	30	37.61 ± 2.29	
	MD8	12	7.5	30	41.63 ± 1.04	
	MD9	12	10	30	42.92 ± 0.67	
	MD7	12	3.5	40	40.98 ± 1.04	
	MD8	12	7.5	40	42.05 ± 1.52	
	MD9	12	10	40	43.25 ± 1.30	

Adapted from corresponding PhD Dissertation^71^.

**Figure 7:**
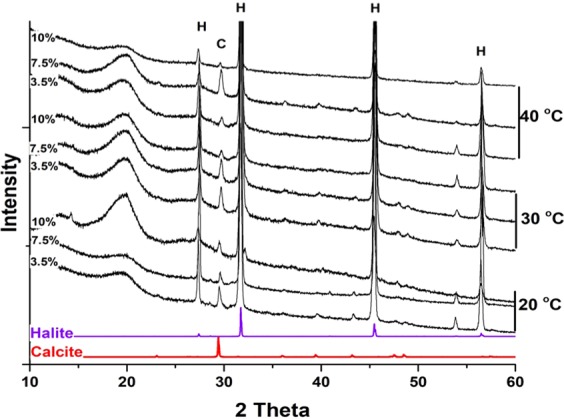
Representative X-ray diffraction patterns of minerals recovered from DF112 and 2141 cultures using media with different salinities and temperatures and a Mg^2+^: Ca^2+^ ratio of 1. Included for comparison are the XRD patterns of calcite and halite standards. C: calcite, H: halite. Modified from corresponding PhD Dissertation^71^.

However, HMC peaks were not detected in the XRD patterns of the minerals recovered at a Mg^2+^: Ca^2+^ ratio of 1; these cultures only produced calcite and halite peaks (Fig. 7).

At Mg^2+^: Ca^2+^ ratio of 6 and 12, the incorporation of Mg^2+^ into the carbonate crystals resulted to be higher in experiments performed at higher NaCl (%w/v) and/or higher temperature (Fig. 6).

Analysis of variance (ANOVA) revealed significant differences in the incorporation of Mg^2+^ among the different conditions for each *Virgibacillus* strain (p-value 0.000). However, the differences between the two *Virgibacillus* strains were not significant (p-value 0.967). The multilinear regression revealed that the effects of the combined three studied parameters were statistically significant with respect to the incorporation of Mg into carbonate minerals (Table 3).

**Table 3:** Results of multiple linear regression.

ANOVA^a^								
Model		Sum of Squares	df	Mean Square	F	Sig.		
1	Regression	2739.440	7	391.349	22.458	0.000^b^		
	Residual	1742.563	100	17.426				
	Total	4482.003	107					
a. Dependent variable: %Mg								
b. Predictors: (Constant), Temp*Salinity*Ratio, Temp*Salinity, Ratio*Temp, Ratio*Salinity, Ratio, Salinity, Temp								
**Model Summary**								
Model	R	R Square	Adjusted R Square	Std. Error of the Estimate	Change Statistics			
					R Square Change	F Change	df1	df2
1	0.782^a^	0.611	0.584	4.17440	0.611	22.458	7	100
**Coefficients**^**a**^								
Model		Unstandardized Coefficients		Standardized Coefficients	t	Sig.	Correlations	
		B	Std. Error	Beta			Zero-order	
1	(Constant)	13.256	2.212		5.992	0.000		
	Temp	0.430	0.049	0.545	8.746	0.000	0.545	
	Salinity	0.913	0.150	0.379	6.084	0.000	0.379	
	Ratio	0.612	0.134	0.285	4.568	0.000	0.285	
	Ratio*Salinity	−0.030	0.050	−0.037	−0.591	0.556	−0.037	
	Ratio*Temp	−0.072	0.016	−0.274	−4.389	0.000	−0.274	
	Temp*Salinity	−0.032	0.018	−0.110	−1.767	0.080	−0.110	
	Temp*Salinity*Ratio	0.002	0.006	0.019	0.312	0.756	0.019	

Indeed, the adjusted R-squared value of 0.584 indicates that 58% of variation in the data is explained by the model, with temperature producing the largest R-squared increase. The standardized coefficients (Beta) values were (0.545, 0.379, and 0.285) for temperature, NaCl (%w/v) and Mg^2+^: Ca^2+^ ratios respectively, suggesting that temperature had the highest impact on Mg incorporation, while the Mg^2+^: Ca^2+^ ratio had the lowest impact. Among the three parameters, the interaction between temperature and Mg^2+^: Ca^2+^ ratio was significant.

Based on microscopic and SEM observations, Raman spectroscopy and confirmation by XRD analysis, no mineral formation was detected in the negative control cultures (i.e., uninoculated medium, autoclaved cells, non-producing bacteria, and medium with no acetate salts).

## Discussion

Aerobic heterotrophs are among the microorganisms that mediate the formation of carbonate minerals. Through their specific metabolic pathways, they create local microenvironments suitable for carbonate precipitation. Possible mechanisms include denitrification (Eqs. 1, 2)^48^ and ammonification (Eqs. 3, 4)^49^.

1 $${{\rm{CH}}}_{2}{{\rm{COO}}}^{-}+4{{\rm{H}}}^{+}2{{\rm{NO}}}_{3}\to 2{{\rm{CO}}}_{2}+{{\rm{N}}}_{2}+3{{\rm{H}}}_{2}{\rm{O}}$$

2 $${{\rm{Ca}}}^{2+}+{{\rm{CO}}}_{2}({\rm{aq}})+2{{\rm{OH}}}^{-}\to {{\rm{CaCO}}}_{3}({\rm{s}})+{{\rm{H}}}_{2}{\rm{O}}.$$

3 $${\rm{NHCO}}\,({\rm{Nitrogen}}\,{\rm{containing}}\,{\rm{matter}})+{{\rm{H}}}_{2}{\rm{O}}\to {{\rm{CO}}}_{2}+{{\rm{NH}}}_{3}$$

4 $${{\rm{NH}}}_{3+}{{\rm{H}}}_{2}{\rm{O}}\to {{\rm{NH}}}_{4}^{+}+{{\rm{OH}}}^{-}$$

The changes occurring in the media, together with the concentration of ions on the cell walls and/or in the EPS create local oversaturation leading to carbonate precipitation^50^.

Temperature is known to favour the precipitation of carbonate minerals by increasing the saturation index^13^. Temperature is also known to help overcome kinetic barriers that otherwise prevent the incorporation of Mg into a crystal. Indeed, dolomite is not difficult to form in laboratory experiments performed at high temperature (e.g.^51,52^). Previous studies using microbial culture experiments showed the existence of a positive correlation between temperature and the amount of Mg incorporated into carbonate minerals^3^. High and low temperatures - beyond optimum range of growth - are a source of stress for the microorganisms^53,54^. In response to such environmental stress, it is known that microbes may react by secreting specific organic molecules – (i.e., EPS) that, in turn, might favour mineral nucleation (i.e.^12,24,55^). For instance, the optimal growth temperature for *Virgibacillus* bacteria genetically close to that used in the present study was reported to be approximately 37 °C, though they can tolerate temperatures between 15–50 °C^56^. With respect to microbially mediated formation of Mg-rich carbonates, the role of temperature might therefore be double: one relates to the thermodynamic and kinetic of the reaction, the other to an ecological stress that causes microbes to increase the production of EPS, which in turn promotes mineral formation. All these considerations are consistent with the results of our experiments carried out at 20, 30, and 40 °C, which showed a positive correlation between temperature and the Mg mol% content of the carbonate mineral (Tables 2, 4 and Fig. 6).

In natural evaporitic environments, all dissolved cations are simultaneously concentrated through progressive evaporation of the seawater^57^. Therefore, salinity is generally positively correlated (although not in a linear manner) with the saturation index of the carbonate minerals. However, in our experiments, the Na^+^ and Cl^−^ concentrations increased independently of the concentrations of Ca^2+^ and Mg^2+^, allowing the separate evaluation of the effect of salinity and the Mg^2+^:Ca^2+^ ratio. The effect of salinity and ionic strength on the incorporation of Mg into the carbonate minerals are not fully resolved, and existing studies show contrasting results. Stephenson *et al*.^58^ reported an inverse relationship between the ionic strength and the Mg content in Mg-rich calcite obtained abiotically. In addition, a theoretical geochemical model for dolomite formation predicted a higher saturation index (SI) at higher salinity^59^. However, various microbial mediation experiments conducted at surface temperatures suggested that high salinity (if not sufficiently high to inhibit microbial growth) generally promotes the formation of Mg-rich carbonates^6,43,50^. This correlation was also clearly observed in our study. For example, the Mg mol% content increased from 27.70 ± 2.00 to 31.36 ± 0.62 to 41.52 ± 1.71 when the NaCl (%w/v) increased from 3.5% to 7.5% to 10% respectively, while the temperature was maintained at 30 °C and the Mg^2+^:Ca^2+^ ratio at 6 (Table 2).

A similar positive correlation as observed between the mol% of Mg and temperature and NaCl (%w/v) was observed for the Mg^2+^:Ca^2+^ ratio. A ratio of 1:1 was not sufficient to form any high-Mg calcite; only Ca-carboante and Mg-calcite with a low mol% of Mg^+2^ were detected. However, a ratio of 12:1 with respect to 6:1 significantly affected the amount of Mg^+2^ incorporated into the mineral. This result is consistant with previous studies proposing that a high Mg^2+^:Ca^2+^ ratio is a key factor for the formation of dolomite in a natural environment^12,29^. For instance, dolomite fromation in the Dohat Faishak sabkha –the site where the microbes used for this study were isolated– has been linked to the precipitation of gypsum, which removes Ca^2+^ from solution and abruptly increases the Mg^2+^:Ca^2+^ ratio (i.e., from ≈3 to ≈12)^29,60^. Our findings using *Virgibcillus* support this hypothesis: a Mg^2+^:Ca^2+^ ratio higher than 1 is vital for the incorporation of Mg^2+^. However, above the tested values of 6 (that corresponds to a free Mg^2+^:Ca^2+^ ratio at the beginning of the experiment of about 5 (see method section)), increasing the temperature resulted to be more important than further increasing the Mg^2+^:Ca^2+^ ratio from 6 to 12.

The observed link between the mol% of Mg into the carbonate mineral and high concentrations of dissolved Na^+^ and Cl^−^ is intuitively not so direct and requires some discussion. An increase in ionic strength does not enhance incorporation of Mg into the carbonate under abiotic conditions but has rather the opposite effect^58^. We therefore propose that to understand this correlation also a biological factor needs to be considered. Several of the most recent studies on microbially mediated formation of dolomite suggest that EPS have key importance in the mineralization process^12,23–25^. EPS include functional groups that, by affecting the kinetics of the nucleation process, were shown to promote incorporation of Mg into carbonate minerals at low temperature. Several studies, indeed, suggest that the chemistry of the cell walls and the associated EPS play a crucial role^24,44,61^. Because EPS is abundantly produced when microbial communities are under ecological stress^62^, we hypothesize that more EPS were formed in the experiments carried out with high concentrations of dissolved Na^+^ and Cl^−^, which in turn resulted in the mediation of carbonate minerals with a higher mol% Mg. EPS compounds bind a variety of divalent cations. Ca^2+^ ions are preferentially absorbed on EPS compared to Mg^2+^ ions due to the fact that the energy used to remove the hydration membrane of Ca^2+^ ions is smaller than that of Mg^2+^ ions. Consequently, this differential binding of Mg^2+^ and Ca^2+^ may result in a Mg^2+^:Ca^2+^ ratio that differs from that of the surrounding environment, influencing the degree of Mg incorporation into the mineral^63^.

The hypothesis that EPS played an important role in our experiments is also consistent with the morphology of the precipitates. Similar morphologies have been described in previous studies on microbial mediation of carbonate minerals^39,64^. It has been proposed that such peculiar rounded shape is mostly controlled by mineral nucleation and growth within a high viscosity solution, which is in turn due to the presence of microbially produced EPS and amino acids^65^. The morphology of the minerals produced in the experiments is therefore not caused by a process whereby a pre-existing grain is progressively coated by concentric layers of authigenic carbonates, as it is the case with ooids – another common facies of spherical carbonates that occur in evaporitic environments^66–69^.

Finally, the observation that different minerals (i.e. calcium carbonate, high magnesium calcite, hydromagnesite) were formed simultaneously in the same pure culture is consistent with mineralization under the influence of heterogenic functional groups present in EPS and/or bacterial cell walls. Organic molecules with different cation-adsorption properties would indeed explain the co/occurrence of minerals with different morphologies and chemical compositions.

## Conclusions

In our experiments that simulate natural sabkhas environment, all the three tested parameters—temperature, NaCl (%w/v), and Mg^2+^:Ca^2+^ ratio—had an impact on microbially mediated formation of high-Mg calcite. Bacterial growth and EPS production, which in turn affected by temperature and NaCl (%w/v), caused an increase in magnesium incorporation into the precipitated minerals. The highest incorporation of Mg^2+^in the carbonate crystals was obtained with a salinity of 10% rather than 3.5% or 7.5% NaCl (%w/v), while calcium carbonates were mostly detected in experiments with 3.5% NaCl (%w/v). With a Mg^2+^:Ca^2+^ ratio of 1, the dominant mineral phase was calcium carbonate; no or rare magnesium calcium carbonates were observed. Increasing the Mg^2+^:Ca^2+^ ratio from 6 to 12 resulted in an increase in Mg^2+^ incorporation into the carbonate crystals. Thus, a high Mg^2+^:Ca^2+^ ratio seems to be important as proposed in most previous models explaining dolomite formation in sabkha environments^12,29^. In summary, at the tested conditions, temperature had the highest impact for the incorporation of Mg^2+^ during the microbially mediated mineralization process, followed by NaCl (%w/v) and Mg^2+^:Ca^2+^ ratio.

Although our experimental approach is far too simple to simulate the complexity of natural environments, the results obtained with two different *Virgibacillus* strains suggest that temperatures, NaCl concentrations and Mg^2+^:Ca^2+^ ratios higher than those of average seawater favour microbially mediated formation of Mg-rich carbonates, which are often considered as potential dolomite precursors.

## Material and Methods

### Bacterial strains

Two *Virgibacillus* strains (*Virgibacillus martsimiure* DF112 and *Virgibacillus* sp. DF2141) and one *Bacillus* strain (*Bacillus licheniformis* DF141) were used in this study. Bacillus and Virgibacillus are aerobic or facultatively anaerobic and spore forming bacteria. They are Gram-staining-positive motile rods, that may occur in single, pairs or chains. The studied strains were previously isolated in our laboratory from core samples collected from the Dohat Faishakh sabkha^5^. Bacterial identification was performed by sequencing the 16 S rRNA gene. The strains used were initially recovered from the established Qatari Mineral Precipitating Strains Bank, where they had been preserved at −80 °C in 30% glycerol. The strains were streaked on solid Luria-Bertani (LB) medium prior to each experiment to obtain viable, fresh, and pure cells.

### Culture media

The nine types of culture media used in this study were labelled MD1–9. The media were composed of 1% (w/v) yeast extract, 0.5% (w/v) peptone, and 0.1% (w/v) glucose. They were supplemented with different concentrations of acetate salts, Ca (C_2_H_3_O_2_)_2_, and (CH_3_COO) _2_Mg 4H_2_O, (Table 4). Mg^2+^:Ca^2+^ molar ratios of 1, 6 and 12 were selected for the experiments. It is important to note that these molar ratios do not correspond to the free ion molar ratios in the growth medium at the beginning of the experiment. With respect to Ca^2+^, Mg^2+^ is indeed preferentially complexed by acetate, which means that the free Mg^2+^ is less than its molar concentration. For example, under the conditions of our experiments, an initial ratio of 6 corresponds approximately to a free Mg^2+^:Ca^2+^ ratio of 5 (modelled in Geochemist’s Workbench 12). The NaCl (%w/v) was adjusted with different concentrations of NaCl (Table 4), and the pH was adjusted to 7 with 0.1 M KOH. Solid media were made by adding 18 g/l agarose to each liquid medium. All the media were sterilized by autoclaving at 121 °C for 20 min.

**Table 4:** Concentrations of acetate salts and NaCl in the nine types of culture media used for the microbial culture experiments.

Medium	Mg^+2^ (mM)	Ca^+2^ (mM)	Mg^2+^:Ca^2+^	NaCl (%)
MD1	9	9	1	3.5
MD2	9	9	1	7.5
MD3	9	9	1	10
MD4	56	9	6	3.5
MD5	56	9	6	7.5
MD6	56	9	6	10
MD7	108	9	12	3.5
MD8	108	9	12	7.5
MD9	108	9	12	10

### Culture experiments at different salinities and temperatures

MD-plates were inoculated with each bacterial strain by surface streaking and incubated aerobically for 30 days at 20 °C, 30 °C, and 40 °C. All cultures were observed regularly under an optical microscope at 40× and 100× magnification to detect the formation of mineral crystals. At the end of incubation periods, the pH was recorded and an average number of crystals/mm^2^ was estimated using optical microscope at 40×. All the experiments were carried out in triplicate and several controls were used. First, *B. licheniformis* DF141, which metabolized acetate salts in a similar manner as the other two strains but could not form mineral crystals under the same experimental conditions, was used as a negative control. Second, controls involving uninoculated plates, autoclaved bacterial cells, and cultures grown on MD medium with no acetate salts were also used as controls. None of the bacterial strains could grow on media with NaCl concentrations above 10%.

### Scanning electron microscopy/energy-dispersive X-ray spectroscopy (SEM/EDS), Raman spectroscopy and X-ray diffraction (XRD) analyses

At the end of the incubation period of each experiment, the crystals were carefully collected using a scalpel and transferred into a 50-ml centrifuge tube, then washed three times with distilled water to remove the salt and impurities by centrifugation at 5000 *g* for 15 min. The minerals were air dried at 40 °C. This procedure did not affect the morphology of the crystals, as verified by optical microscopy and SEM before and after crystal recovery. Dried samples were used for the SEM/EDS, Raman spectroscopy and XRD analyses.

SEM images were obtained using a FEI Quanta 200 Environmental Scanning Electron Microscope (ESEM) with a resolution of 5 nm and a magnification of 200,000× equipped with an energy-dispersive X-ray microanalysis system (model 2011-Netherlands). The EDS was carried out following “ASTM standard method E1508–12a”, with spot size 5 at an accelerating voltage of 20 kV and a 4% error.

The recovered crystals were placed and oriented on the stage of an Olympus BHSM microscope, equipped with 10×, 20× and 50× objectives that was part of a DXR Thermo-Scientific DXR Raman imaging microscope, which includes a monochromator, a filter system and a charge coupled device (CCD). Raman spectra were excited by a 532 nm laser at a nominal resolution of 2 cm^−1^ in the range between 50 and 3500 cm^−1^. The Omnic D/S software was used to perform spectral baseline correction/adjustment and smoothing.

To determine their mineralogical composition, particles were selected and targeted for discrete X-ray analysis. The bulk mineralogical composition was performed using a PANalytical- multipurpose Empyrean X-ray diffractometer. The analysis of XRD spectra was carried out using MATCH! Version 3.5.2.104, CRYSTAL IMPACT, Kreuzherrenstr. 102, 53227 Bonn, Germany.

The Mg mol% of the carbonate minerals formed in the experiments was calculated using the formula of Goldsmith *et al*.^70^, which is based on the position of the (d104) peak in the XRD pattern.

### Statistical analysis

All the experiments were carried out in triplicates. The significance of differences between conditions was analysed using a one-way analysis of variance (ANOVA). Multilinear regression analysis was performed to estimate the association between the three independent variables (temperature, NaCl (%w/v) and Mg^2+^:Ca^2+^ ratios) and the mol% Mg and to determine which predictor variable(s) are the most important. Statistical analyses were carried out at the 95% confidence level using the software IBM SPSS Statistics Version 24.
